# Drinking Water with Uranium below the U.S. EPA Water Standard Causes Estrogen Receptor–Dependent Responses in Female Mice

**DOI:** 10.1289/ehp.9910

**Published:** 2007-09-14

**Authors:** Stefanie Raymond-Whish, Loretta P. Mayer, Tamara O’Neal, Alisyn Martinez, Marilee A. Sellers, Patricia J. Christian, Samuel L. Marion, Carlyle Begay, Catherine R. Propper, Patricia B. Hoyer, Cheryl A. Dyer

**Affiliations:** 1 Department of Biological Sciences, Northern Arizona University, Flagstaff, Arizona, USA; 2 Department of Physiology, College of Medicine, University of Arizona, Tucson, Arizona, USA

**Keywords:** depleted uranium, endocrine disruption, estrogen, estrogen receptor, female reproduction, heavy metal, Navajo reservation

## Abstract

**Background:**

The deleterious impact of uranium on human health has been linked to its radioactive and heavy metal–chemical properties. Decades of research has defined the causal relationship between uranium mining/milling and onset of kidney and respiratory diseases 25 years later.

**Objective:**

We investigated the hypothesis that uranium, similar to other heavy metals such as cadmium, acts like estrogen.

**Methods:**

In several experiments, we exposed intact, ovariectomized, or pregnant mice to depleted uranium in drinking water [ranging from 0.5 μg/L (0.001 μM) to 28 mg/L (120 μM).

**Results:**

Mice that drank uranium-containing water exhibited estrogenic responses including selective reduction of primary follicles, increased uterine weight, greater uterine luminal epithelial cell height, accelerated vaginal opening, and persistent presence of cornified vaginal cells. Coincident treatment with the antiestrogen ICI 182,780 blocked these responses to uranium or the synthetic estrogen diethylstilbestrol. In addition, mouse dams that drank uranium-containing water delivered grossly normal pups, but they had significantly fewer primordial follicles than pups whose dams drank control tap water.

**Conclusions:**

Because of the decades of uranium mining/milling in the Colorado plateau in the Four Corners region of the American Southwest, the uranium concentration and the route of exposure used in these studies are environmentally relevant. Our data support the conclusion that uranium is an endocrine-disrupting chemical and populations exposed to environmental uranium should be followed for increased risk of fertility problems and reproductive cancers.

Uranium, the heaviest naturally occurring element, is valued for its radioactive properties. Development of nuclear weapons in the 1940s fueled the U.S. government’s desire to become independent of foreign sources of U (Ball 1993; Moure-Eraso 1999; Panikkar and Brugge 2007). The U “boom” in the southwestern United States lasted from the early 1950s until the market collapsed in 1971, when the U.S. government ceased being the sole purchaser of U ore (Brugge and Goble 2002).

The majority of U mining/milling occurred in the Four Corners region of the United States where the Navajo Reservation is located. The Navajo Abandoned Mine Lands (AML) agency reclaims abandoned uranium mines (AUMs) under the authority and with funding from the Surface Mining Control and Reclamation Act of 1977 (Office of Surface Mining 1977). The Navajo AML agency has estimated that there are approximately 1,300 AUMs throughout the 27,000 square miles of the Navajo Nation (U.S. EPA 2004). About 50% of AUMs have been reclaimed [U.S. Environmental Protection Agency (EPA) 2004]. Unremediated AUMs enabled U to disperse into air, soil, water, and the food chain (Brugge and Goble 2002). A present-day example of unregulated U mining/milling is the Atlas Corporation Moab Uranium Mill Tailing (Moab, UT). Nearly 10,000 gallons of U-contaminated water seeps into the Colorado River daily (Oak Ridge National Laboratory 1998), and the adjacent surface water concentration of uranium is > 5 mg/L (Department of Energy 2005).

The largest American Indian reservation in the United States is the Navajo Nation, which is divided into 110 political units called Chapters. Within 33 Chapters, the U.S. EPA surveyed 226 water sources. Of these, 90 water sources were contaminated with U above the U.S. EPA safe drinking water level of 30 μg/L (0.126 μM). The U levels found in contaminated water sources ranged from 33.3 to 1,131 μg/L, with the highest concentration being 38 times the safe drinking water level (U.S. EPA 2004). The surveyed water sources were stock tanks, wells, and springs. Chapter officials identified the water sources as providing drinking water for residents without running water (U.S. EPA 2004). According to the 2000 U.S. census (2006), > 175,000 people live on the Navajo Reservation. At least half of these residents haul water from the nearest water source for household use (i.e., drinking water, cooking, and clothes laundering), making it a certainty that many Navajo Nation residents are exposed to unsafe levels of U.

The toxicity of U is due to its radioactive and chemical properties (Brugge et al. 2005; Taylor and Taylor 1997). U inhalation and/or ingestion leads to malignant and non-malignant respiratory diseases, stomach and kidney cancer, kidney failure, and leukemia (Brugge et al. 2005; Roscoe et al. 1995). U’s effect on the reproductive system was examined in early studies with rats fed high doses of 2% uranyl nitrate (UN). U exposure caused significant weight loss in dams, fewer litters, and fewer pups per litter (Maynard and Hodge 1949). When female rats were returned to chow diet without UN, they regained the lost body weight, but a reduction in the number of litters and pups per litter persisted, suggesting that the ovaries had been permanently damaged (Maynard and Hodge 1949). Female mice treated with uranyl acetate by gavage through gestation, parturition, and nursing had an increased number of dead young per litter (Paternain et al. 1989). It is likely that the high doses of U in these studies led to reproductive toxicity (Domingo 2001; Hindin et al. 2005).

Heavy metals exhibit estrogenic properties (Dyer 2007). Several heavy metals stimulate proliferation of MCF-7 human breast cancer cells (Brama et al. 2007; Choe et al. 2003; Martin et al. 2003; Martinez-Campa et al. 2006). Cadmium interacts with estrogen receptor-α(ER-α) (Brama et al. 2007; Martin et al. 2003) and binds to the ligand-binding domain of ER-α in cultured cells (Stoica et al. 2000). Cd stimulates estrogenic responses *in vivo* (Alonso-Gonzalez et al. 2007; Johnson et al. 2003). Ovariectomized rats injected with Cd had increased uterine weight, accelerated mammary gland growth/development, and accelerated vaginal opening (VO) (Johnson et al. 2003). Cd-induced estrogen-like responses were prevented by the antiestrogen ICI 182,780. Cd inhibits transcriptional activity of estradiol-activated rainbow trout ER in recombinant yeast (Guével et al. 2000). Cd treatment stimulates breast cancer cell proliferation by activating ER-α–dependent Akt (protein kinase B), Erk1/2 (extracellular signal-regulated kinase), and platelet-derived growth factor receptor-α (Brama et al. 2007). Although these studies demonstrate the estrogen activity of Cd, it should be noted that Silva et al. (2006) reported that Cd lacks estrogenic activity in the yeast estrogen screen assay, MCF-7 cell proliferation, or the E-SCREEN assay, and also failed to induce Src, Erk1, and Erk2 phosphorylation. In the present study we tested whether depleted U added to drinking water caused responses in the female mouse reproductive tract like those caused by the potent synthetic estrogen diethylstilbestrol (DES).

## Materials and Methods

### Animals

We performed U exposure in intact female mice using 28-day-old immature B6C3F_1_ mice (Harlan, Indianapolis, IN). For *in utero* U exposure experiments, we used 48-day-old male and female B6C3F_1_ mice (Harlan). We used ovariectomized 28-day-old C57Bl/6J mice (The Jackson Laboratory, Bar Harbor, ME) for the prepubertal U and DES exposure experiments. Mice were housed with a 12:12 hr light/dark cycle and received water and food *ad libitum*. Control tap water tested for U using kinetic phosphorescence analysis, as described by Hedaya et al. (1997), was below the limit of detection (< 2 μg/L or < 8 pM). All protocols were approved by the University of Arizona or Northern Arizona University Institutional Animal Care and Use Committees. All mice were treated humanely with regard for alleviation of suffering in accordance with the NIH *Guide for the Care and Use of Laboratory Animals* (Institute of Laboratory Animal Research, 1996).

### Treatments

Animals were treated with UN hexahydrate (depleted U) (Sigma Chemical Co., St. Louis, MO) in drinking water.

#### Study 1: Impact of U exposure on ovarian follicle populations

##### Experiment 1.1: U exposure in immature mice

Mice were exposed to UN in their drinking water at milligram per liter doses based on a study using rats (Gilman et al. 1998). Immature 28-day-old B6C3F_1_ mice drank water containing UN at 0.5, 2.5, 12.5, and 60.0 mg/L (1, 5, 25, and 120 μM, respectively; *n* = 9–10 mice per group). After 30 days, we analyzed ovaries for changes in follicle populations.

##### Experiment 1.2: Gestational and *in utero* U exposure in dams and female pups

For *in utero* exposure, mice were given water containing UN at 0.5, 2.5, 12.5, or 60 μg/L (0.001, 0.005, 0.025, or 0.120 μM U, respectively) for 30 days prior to breeding. U dose was reduced a thousandfold to micrograms per liter to correspond to environmentally relevant concentrations. Mice were paired for breeding, and males were removed when females had vaginal plugs. Females continued to drink U-containing water at the above doses through gestation. On the day of birth, dams (*n* = 5 mice per treatment group) and female pups (*n* = 7–9 pups per treatment group) were euthanized and the ovaries collected for histology.

#### Study 2: Impact of U exposure on the female reproductive tract in the absence of endogenous estrogen

##### Experiment 2.1: U exposure in ovariectomized mice

For this study we used C57Bl/6J mice because of strain sensitivity to estrogen in the uterotrophic assay (Ashby et al. 2003). We also anticipated the use of genetically manipulated mice (e.g., ER-α knockout mice) on this genetic background (Lubahn et al. 1993). C57Bl/6J mice were ovariectomized at 28 days of age to remove the endogenous source of estrogen before VO. Seven days postsurgery, ovariectomized and intact mice were given tap water or water containing 0.19 μM DES or 0.06, 0.12, 1.20, or 12.00 μM U for 30 days (*n* = 5–6 mice per treatment group).

##### Experiment 2.2: Other estrogen-like effects of UN and dependence on ER activation

Mice ovariectomized at 28 days of age were exposed to drinking water containing U or DES at the aforementioned concentrations for 10 days beginning at 50 days of age. Some mice (*n* = 6–7 mice per group) concurrently received daily intraperitoneal (i.p.) injections of either sesame oil vehicle or 500 μg/kg ICI 182,780 (Tocris Coolson Ltd., Avonmouth, UK). Mice were examined daily at the same time for VO and cytology.

### Tissue collection and histology

After exposure to DES or U, mice were euthanized and organs were collected for necropsy. Uteri were removed by dissecting inferior to the Fallopian tubes and superior to the vagina. Wet weights of ovary, uterus, kidney, liver, and spleen were normalized to total body weight. Uterine tissues were fixed in Bouin’s solution, embedded in paraffin, and serially sectioned every 9 μm; every 10th section was mounted on slides. Tissue sections were deparaffinized in Citrasolve (Sigma Chemical Co.) and dehydrated in a series of ethanol baths. We used a Zeiss 435 VP scanning electron microscope and LEO32 V02.01 software (Carl Zeiss SMT Inc., Peabody, MA) to measure the height of uterine luminal epithelial cells. Forty measurements were randomly collected from each individual uterus.

Ovaries were trimmed of adhering tissue and fat and then fixed in Bouin’s solution. They were transferred to 70% ethanol, embedded in paraffin, serially sectioned (5 μm), mounted, and stained with hematoxylin and eosin. Nuclei of oogonia and primordial, small primary, large primary, secondary or growing, and healthy antral and atretic follicles were identified and counted in adult ovary every 20th section, and in pup ovary every 12th section (Mayer et al. 2004).

### Statistical analyses

Oogonia and follicle numbers were determined in ovaries from individual mice and averaged. The means in control versus exposed mice were analyzed for significant differences by one-way analysis of variance (ANOVA) with significance set at *p* < 0.05. We used Tukey-Kramer post hoc tests where appropriate. For mice exposed for 10 and 30 days, organ weights were determined for each individual within each experiment and averaged for each exposure group. In the 30-day–exposure group, uterine luminal epithelial cell height measurements were collected from individual mice and averaged for each exposure group. Additionally, in the 10-day–exposure group, VO was determined for each individual and averaged for the exposure group. The means for control versus exposed mice for organ weights, uterine epithelial cell height, and VO were analyzed for significant differences by one-way ANOVA with significance set at *p* < 0.05. We used Dunnet’s post hoc test where appropriate. The means of uterine weights in controls or in mice exposed to ICI 182,780, U, or DES were analyzed by two-way ANOVA with significance set at *p* < 0.05. Persistent presence of cornified vaginal cells was determined for each individual mouse in the 10-day–exposure group. Presence and absence of cornified cells was analyzed by chi-square test with significance set at *p* < 0.05. Statistical significance of persistent presence of cornified cells was analyzed by Fisher’s exact test with significance set at *p* < 0.05.

## Results

### Study 1: Impact of U exposure on ovarian follicle populations

#### Experiment 1.1: U exposure in immature mice

Experiment 1.1: showed that U targets early stage ovarian follicles. As shown in Table 1, there were significantly fewer large primary follicles at 0.5 and 2.5 mg/L UN and significantly more secondary or growing follicles at 12.5 mg/L UN. However, we found no significant increase in the number of atretic follicles or decrease in healthy follicles. Because UN exposure caused a selective change in ovarian follicle populations and because there were more growing follicles at 12.5 mg/L UN, the changes could not be caused by heavy metal toxicity.

This experiment also showed that U does not lead to overt organ toxicity. We found no gross anomalies in any major organs, and body weight did not significantly change with UN exposure at any concentration. As shown in Table 2, kidney weight was significantly reduced at doses of 2.5 and 60.0 mg/L UN, but this was not surprising given the nephrotoxicity of U (Brugge et al. 2005; Taylor and Taylor 1997). These data support the conclusion that there was no systemic UN-mediated toxicity.

We found an interesting, but not statistically significant, trend of increased uterine weight at 12.5 and 60.0 mg/L UN (Table 2). We did not determine estrous cycle stage in mice at sacrifice, thus uterine weights could not be grouped relative to stage.

#### Experiment 1.2: Gestational and *in utero* U exposure in dams and female pups

**Figure 1:**
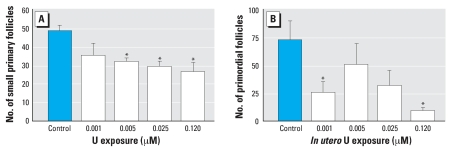
Effects of UN at 0.5, 2.5, 12.5, or 60 μg/L (0.001, 0.005, 0.025, or 0.120 μM U, respectively) on dam follicle populations and *in utero* exposed pup ovary primordial follicles. B6C3F_1_ dams were exposed to control tap water or U in drinking water for 30 days before mating and through gestation. Ovaries from dams (*A*) and pups (*B*) were removed on the day of birth. Values shown are mean ± SE (*n* = 7–11). *Significantly different compared with controls (*p* < 0.05, ANOVA).

Experiment 1.2 showed that *in utero* uranium exposure reduces pup ovary primordial follicles. As shown in Figure 1A, mice exposed to UN for 30 days before mating and through gestation had a significant reduction of small primary follicles at UN concentrations of 0.005, 0.025, and 0.120 μM compared with control mice. All other follicle populations, including primordial, secondary/growing, healthy, and atretic, were unchanged (data not shown). Neonatal mouse ovaries have only oogonia and primordial follicles. We found no difference in the number of pup ovary oogonia among control and UN exposure groups (data not shown). Primordial follicle numbers were reduced in ovaries of pups whose dams consumed water with 0.001- or 0.120-μM UN, compared with primordial follicles in pup ovaries from dams drinking control tap water (Figure 1B).

### Study 2: Impact of U exposure on the female reproductive tract in the absence of endogenous estrogen

#### Experiment 2.1: U exposure in ovariectomized mice

**Figure 2:**
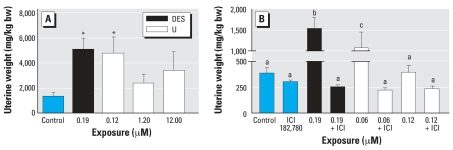
Effect of UN or DES alone and in combination with ICI 182,780 on uterine weight in ovariectomized C57Bl/6J mice. (*A*) Uteri were removed after 30 days of exposure, and wet weights were recorded and normalized to body weight; values shown are mean ± SE (*n* = 5–6). (*B*) Uteri were removed after 10 days of exposure, and wet weights were recorded and normalized to body weight; values shown are mean ± SE (*n* = 6–7). Different letters (a, b, c) indicate significant differences among exposure groups (*p* < 0.005). *Significantly different compared with other exposure groups (*p* < 0.001).

Experiment 2.1 showed that UN exposure induces estrogen-like changes in uterine morphology and histology. Mice exposed to UN or DES had significantly increased uterine weight at 0.120 μM U and 0.19 μM DES, 3.6 and 3.8 times greater, respectively, compared with mice drinking control tap water (Figure 2A). We normalized uterine weights to body weights, which were unchanged across treatment groups. Uterine weights were not increased in ovary-intact, age-matched mice that drank U-containing water (data not shown).

#### Experiment 2.2: Other estrogen-like effects of UN and their mediation through ER activation

Experiment 2.2 showed that UN-mediated estrogen-like actions are blocked by concomitant exposure to an ER antagonist. To determine if the U-mediated uterotrophic response was dependent on ER activation, ovariectomized mice drinking UN-containing water were injected daily with the antiestrogen ICI 182,780. In a pilot experiment, we determined that 10 days of exposure to UN in drinking water caused a significant increase in uterine weight compared with mice drinking tap water (data not shown). Ten days of concomitant ICI 182,780 treatment blocked both UN- and DES-mediated increases in uterine weights (Figure 2B): 0.06 μM U alone, 1,070 ± 386 mg/kg total bw; 0.06 μM U plus ICI 182,780, 220 ± 28.1 mg/kg total bw; 0.19 μM DES alone, 1,530 ± 282 mg/kg total bw; 0.19 μM DES plus ICI 182,780, 252 ± 24.7 mg/kg total bw. Uterine weights of control mice were not significantly different from controls treated with ICI 182,780 (Figure 2B).

**Figure 3:**
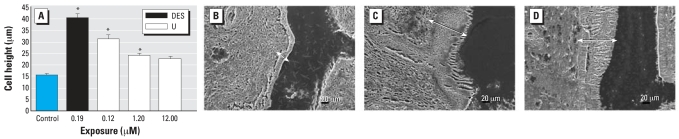
Uterine luminal epithelial cell growth in ovariectomized C57Bl/6J mice stimulated by UN or DES in drinking water for 30 days. (*A*) Cell height in uteri collected and prepared for scanning electron microscopy; values shown are mean ± SE (*n* = 5 uteri at 40 measurements from each tissue). Representative scanning electron microscopy images at the same magnification of uterine epithelial cell layers from tap water control (*B*), 0.19 μM DES (*C*), or 0.12 μM U (*D*). Arrows highlight epithelial cell height in DES-exposed (*C*) and U-exposed (*D*) ovariectomized mice. *Significantly different compared with control (*p* < 0.0001).

One aspect of the uterotrophic response to estrogen is proliferation of the epithelial cell lining of the uterus (Kang et al. 1975; O’Brien et al. 2006). Uterine epithelial cell height was significantly greater in mice drinking water containing U or DES for 30 days (Figure 3A); 0.120 μM U, 31.01 ± 1.89 μm; 1.20 μM U: 23.79 ± 0.93 μm; 0.19 μM DES, 40.2 ± 1.85 μm; controls, 15.24 ± 0.77 μm. Figures 3B (control), 3C (0.19 μM DES), and 3D (0.12 μM U) show scanning electron micrographs illustrating changes in uterine luminal epithelial cell height. Arrows in in Figure 3C and 3D indicate pseudostratified columnar morphology typical of proliferating epithelial cells due to DES or UN exposure, respectively.

##### Effects of U on VO and vaginal cell cornification

**Figure 4:**
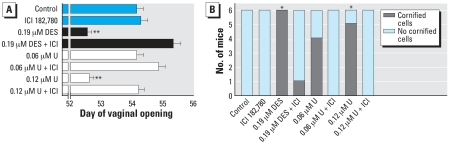
Effect of UN in drinking water on VO and presence of cornified vaginal cells. Ovariectomized C57Bl/6J mice (50 days of age) were exposed to control tap water, 0.19 μM DES, or 0.06 or 0.12 μM U for 10 days, or one of these doses plus vehicle or 500 μg/kg ICI 182,780 in vehicle. (*A*) Mice were examined daily for VO from 50 days of age to the day of vaginal opening; values shown are mean day of VO ± SE (*n* = 6–7). (*B*) Vaginal cell cornification determined from vaginal smears collected daily; the presence and absence of vaginal cornified cells were analyzed by chi-square test (*p* < 0.05). *Statistically significant compared with control (*p* < 0.05 by Fisher’s exact test). **Significantly different from control (*p* < 0.001).

Estrogen and endocrine-disrupting chemicals (EDCs) accelerate VO in mice (Markey et al. 2001). Ovariectomized mice exposed to 0.12 μM UN or 0.19 μM DES exhibited significantly accelerated VO (both at 52.5 days), compared with control mice (54 days) (Figure 4A). UN- or DES-mediated acceleration of puberty onset, as indicated by day of VO, was prevented by concomitant treatment with the antiestrogen ICI 182,780 (Figure 4A).

Another indication of estrogenic influence on the female reproductive tract is the persistent presence of cornified cells in vaginal smears (Gordon et al. 1986). As shown in Figure 4B, mice exposed to 0.06 μM UN (4 mice) or 0.12 μM UN (5 mice), or 0.19 μM DES (6 mice) had persistent presence of cornified vaginal cells compared with control mice (0 mice). Coincident treatment with ICI 182,780 prevented the presence of cornified vaginal cells (0.06 μM UN, 0 mice; 0.12 μM UN, 0 mice; 0.19 μM DES, 1 mouse).

## Discussion

The major contribution of the present study is the discovery that U, similar to other heavy metals, has estrogenic activity (Alonso-Gonzalez et al. 2007; Brama et al. 2007; Choe et al. 2003; Dyer 2007; Johnson et al. 2003; Martin et al. 2003; Martinez-Campa et al. 2006). To our knowledge, this has not been demonstrated before. Immature animals exposed to U in drinking water had increased uterine weight and uterine luminal epithelial cell growth, selective reduction of ovarian primary follicles but more growing follicles, accelerated VO, and persistent presence of cornified vaginal cells. U-mediated responses were blocked by coadministration of the antiestrogen ICI 181,720, indicating that an activated ER was necessary. In addition, transplacental exposure to U caused fewer primordial follicles in developing pup ovaries. These observations support the conclusion that U acts like estrogen in the female mouse reproductive tract.

U caused estrogenic responses at or below the U.S. EPA safe drinking water level of 30 μg/L (0.126 μM) (U.S. EPA 2006). The U.S. EPA safe drinking water level equals the concentration of elemental U and is 47.4% of UN dissolved in water. Therefore, the highest UN concentration of 60 mg/L equals 28 mg/L of elemental U. At first, we used milligram per liter amounts of UN in the drinking water because we expected U to cause ovarian chemical toxicity as previously reported (Maynard and Hodge 1949). Unexpectedly, at milligram per liter concentrations, U targeted only large primary follicles, causing a reduction in their number but an increase in growing follicles. At the same time, there was a trend of increasing uterine weight with increasing U dose. These results led us to determine whether U could mimic estrogen’s effects on the female reproductive system. Subsequently, we analyzed uterotrophic responses in ovariectomized mice using environmentally relevant U concentrations. We observed significant effects of U on the female reproductive system at or below the U.S. EPA safe levels.

The U levels used in these experiments are well within the range of U concentrations measured in numerous water sources on the Navajo Reservation, where concentrations > 1 mg/L have been reported (Brugge and Goble 2002; U.S. EPA 2004). The Navajo Reservation is a vast expanse of primarily rural and open range land. At least half of the households on the Navajo Reservation rely on water hauled from the nearest source for household use (U.S. Census 2006). Given the frequency of water supplies with unsafe U content, there is no doubt that many of the 175,000 residents living on the Navajo Reservation are exposed to hazardous levels of U in their water (Brugge et al. 2007; Pasternak 2006).

Adult mice exposed to U while immature had fewer primary follicle populations but more secondary follicles. 17β-Estradiol (E_2_) inhibits mouse oocyte nest breakdown and follicle assembly (Chen et al. 2007). U, mimicking E_2_ action, may have reduced follicle assembly leading to fewer primary follicles. Dam ovaries had fewer small primary follicles at a 1,000-fold lower U concentration than did the adult nonpregnant mice, which had no significant decrease in primary follicles. The pregnant dam ovaries may have been more sensitive to U because of an up-regulation of ERs that occurs during pregnancy (Spong et al. 2000). Estrogen prevents early follicle assembly (Chen et al. 2007) but stimulates secondary or growing follicles (Drummond 2006). U exposure may have reduced primary follicle populations and stimulated growing follicles via its estrogen-like activity.

Developing embryos are exquisitely sensitive to chemical influences. U concentrations of 0.001 or 0.120 μM in the dams’ drinking water led to a significant reduction in the number of primordial follicles in pup ovaries. Gestational DES exposure is linked to fewer primordial follicles in pups, resulting in fewer ovulated ova (McLachlan et al. 1982). The long-term consequence of fewer primordial follicles would lead to accelerated ovarian failure, resulting in an earlier menopause onset (Chen et al. 2007). The change in pup ovary primordial follicles with uranium dose was an inverted U-shaped curve. Inverted U-shaped curves are seen in responses resulting from *in utero* exposure to E_2_ (Welshons et al. 2003).

The rodent uterotrophic assay is used to identify putative EDCs. Exposure to chemicals with estrogenic activity are analyzed in immature rodents or ovariectomized mature rodents (Markey et al. 2001; Owens and Ashby 2002; Padilla-Banks et al. 2001). In our first experiment, the mice were immature at the outset but became sexually mature during the 30-day exposure to U. These mice exhibited a trend of increased uterine weight. If these mice had been examined for estrous stage at sacrifice, the uterine weights could have been grouped by stage, possibly enabling the trend to reach statistical significance. We used ovariectomized mice to avoid the confounding effect of estrous cycling to test whether UN caused uterotrophic responses.

The uterotrophic assay measures the consequences of three coordinated responses to estrogen or a chemical that acts like estrogen: epithelial cell growth, hyperemia, and fluid accumulation or imbibition (O’Brien et al. 2006). DES stimulation of uterine epithelial cell growth, in addition to employing classical ER-α, may also use tethered or nonclassical pathways to induce mitogenic uterine responses (O’Brien et al. 2006). This suggests that U does not need to directly activate the classical ER for uterine epithelial cell growth.

The U dose response was not monotonic in either the uterotrophic assay or in increased uterine epithelial cell height. Many EDCs elicit low-dose responses resulting in U-shaped or inverted U-shaped dose–response curves (Myers and Hessler 2007; Welshons et al. 2003). Nonmonotonic response occurs when a xenoestrogenic compound exerts direct effects by mimicking estradiol or indirect effects by interfering with ERs or estradiol production and metabolism. Further, xenoestrogenic responses may activate or inhibit different genes at various doses, which may result in different outcomes for target end points examined at the same time points (Coser et al. 2003).

Mice exposed to U for 30 days had a more pronounced uterotrophic response than mice exposed for 10 days. This raises questions about how U may be getting into cells/tissues and by which mechanism U interacts with the ER. U enters brain endothelial cells (Dobson et al. 2006), and via specialized transport it enters polarized epithelial LLC-PK_1_ cells (Muller et al. 2006). Vidaud et al. (2007) examined the possibility of apotransferrin transporting U into the cell. U binds to transferrin, but conformational changes do not enable transferrin receptor recognition of the U-transferrin complex, ruling out this pathway for U to enter the cell. Other ways that U may enter the cell have not been investigated: divalent metal transporter-1 (DMT-1) or calcium channels. DMT-1 functions to transport iron and other metal ions across the plasma membrane, and is ubiquitous in plants, insects, microorganisms, and vertebrates (Mims and Prchal 2005). U displaces calcium in the bone matrix (Neuman et al. 1949); therefore, it is plausible that U may use calcium channels to enter the cell. The manner and rate by which U gets into the cell may be impeded by U speciation or tissue concentration, which could result in delayed responses, as we observed with uterine weight changes after 10-day exposure compared with 30-day exposure.

Similar to DES, U accelerated VO and stimulated persistent vaginal cornified cells, which represents a constant estrus state elicited by estrogen. U-stimulated uterine and vaginal responses were blocked by ICI 182,780, indicating that ER activation was necessary but not sufficient for U to act. We have yet to define the molecular mechanisms of action by which U evokes estrogenic responses. It is possible that U may elicit estrogen-like responses as Cd is reported to, by binding the ligand binding domain of the ER (Stoica et al. 2000). As mentioned above, U estrogenic stimulation may be the result of U binding some other factor whose responses are “tethered” to the ER pathway, resulting in cross-talk that induces estrogenic responses. In summary, the stimulatory effects of U on cells of the the ovary, uterus, and vagina suggest that U acts like estrogen in the female reproductive system and is an EDC.

There are few reports relating environmental U exposure to reproductive health outcomes in the Four Corners region. However, in one study, a statistically significant relationship was found between birth defects and the mother’s proximity to U tailings (Shields et al. 1992). In another study, the incidence of reproductive or gonadal cancer in New Mexico Native American children and teenagers is 8-fold greater than that in age-matched non-Native American individuals (Duncan et al. 1986). Environmental estrogens such as DES or bisphenol A may contribute to occurrence of reproductive anomalies and cancer later in life (Maffini et al. 2006; Newbold et al. 2006). Given our results that U is an EDC, health problems may result from inappropriate concentration or timing of exposure to this estrogen mimic.

## Figures and Tables

**Table 1:** Effects of UN exposure on specific ovarian follicle populations (follicle counts per ovary; mean ± SE) in B63CF_1_ mice exposed to UN in drinking water for 30 days.

		UN (mg/L)			
Follicle type	Control (U < 0.002 mg/L)	0.5	2.5	12.5	60.0
Primordial	65.55 ± 7.05	53.80 ± 8.26	37.88 ± 7.01	57.60 ± 13.29	61.60 ± 12.76
Small primary	26.22 ± 2.50	19.40 ± 3.03	18.56 ± 2.94	32.00 ± 3.51	21.78 ± 2.81
Large primary	12.66 ± 0.69	6.50 ± 1.17*	7.44 ± 1.27*	12.00 ± 1.51	9.11 ± 0.65
Secondary or growing	26.44 ± 1.08	24.20 ± 2.09	21.22 ± 1.85	33.30 ± 1.92*	26.78 ± 0.81
Healthy antral	31.22 ± 2.56	31.00 ± 3.49	28.22 ± 3.71	29.00 ± 2.39	23.11 ± 2.78
Atretic antral	17.22 ± 1.37	15.50 ± 2.37	11.44 ± 1.70	16.00 ± 3.26	12.53 ± 1.37

*n* = 6 per group.

* Significantly different from control (*p* < 0.05, Tukey-Kramer post hoc test).

**Table 2:** Effects of UN exposure on body weight and tissue weight in B63CF_1_ mice exposed to UN in drinking water for 30 days.

Treatment	Body weight	Ovary	Uterus	Liver	Adrenal	Kidney	Spleen
Control (< 2 μg/L U)	100.0	100.0	100.0	100.0	100.0	100.0	100.0
U (mg/L)							
0.5	101.2	77.5	97.1	94.2	95.5	96.0	104.0
2.5	100.4	72.5	81.8	94.4	88.4	91.7*	89.9
12.5	104.1	73.9	115.9	99.2	120.8	100.9	103.6
60.0	104.6	62.4	127.8	110.6	108.5	94.2*	109.8

Tissue weights are expressed as a percent of control values normalized to total body weight.

* Significantly different from control (*p* < 0.05).
